# Triggerfish uses chromaticity and lightness for object segregation

**DOI:** 10.1098/rsos.171440

**Published:** 2017-12-20

**Authors:** Laurie Mitchell, Karen L. Cheney, Fabio Cortesi, N. Justin Marshall, Misha Vorobyev

**Affiliations:** 1 Institute of Marine Science, University of Auckland, Private Bag 92019, Auckland, AKL 1142, New Zealand; 2 School of Optometry and Vision Science, University of Auckland, Private Bag 92019, Auckland, AKL 1142, New Zealand; 3 School of Biological Sciences, University of Queensland, St Lucia, Brisbane, Queensland 4072, Australia; 4 Queensland Brain Institute, University of Queensland, St Lucia, Brisbane, Queensland 4072, Australia

**Keywords:** colour vision, generalization, chromatic cues, visual segregation, reef fish

## Abstract

Humans group components of visual patterns according to their colour, and perceive colours separately from shape. This property of human visual perception is the basis behind the Ishihara test for colour deficiency, where an observer is asked to detect a pattern made up of dots of similar colour with variable lightness against a background of dots made from different colour(s) and lightness. To find out if fish use colour for object segregation in a similar manner to humans, we used stimuli inspired by the Ishihara test. Triggerfish (*Rhinecanthus aculeatus*) were trained to detect a cross constructed from similarly coloured dots against various backgrounds. Fish detected this cross even when it was camouflaged using either achromatic or chromatic noise, but fish relied more on chromatic cues for shape segregation. It remains unknown whether fish may switch to rely primarily on achromatic cues in scenarios where target objects have higher achromatic contrast and lower chromatic contrast. Fish were also able to generalize between stimuli of different colours, suggesting that colour and shape are processed by fish independently.

## 1. Introduction

The survival of visually adept animals depends on their ability to detect and identify prey, predators and conspecifics that are often concealed by shadows and/or camouflaged by disruptive patterns [1–3]. Colour vision enhances the ability of animals to detect objects and it has been suggested that colour vision originally evolved as an adaptation for object detection in conditions of changing and patchy illumination [4,5]. Different animals solve these problems in different ways depending on the constraints imposed both by the external light environment of their specific habitat and the internal neural processing capability provided by the brain.

Humans group the components of visual patterns according to their colour, and perceive colours largely separate from shape [6,7]. Both these features of our perception can be explained as a by-product of certain aspects of processing of visual information in the human retina and brain [8]. Perceptual grouping is the substance of Gestalt psychology, and in humans, the grouping is explained by complex cortical processing that allows us to perceive a whole object that is different from the sum of its elements [9,10]. The separate perception of colour and shape can, to an extent, be attributed to parallel processing of visual information in the human brain [7]. On the other hand, perceptual grouping on the basis of colour, and the independent processing of colour and shape can be explained as an adaptation for the optimal detection and identification of objects in natural conditions. The separate processing of shape and colour is advantageous for object identification, because the shape alone permits us to recognize an object, while colour conveys information about its quality, such as ripeness of a fruit [4,6,11]. The two information streams are combined during the final decision.

For humans, chromaticity has higher saliency than lightness and accordingly we group components of visual patterns predominantly on the basis of their chromaticity [4]. While we perceive lightness largely separately from chromaticity, animals may or may not perceive lightness separately from chromatic aspects of colour. In animals, the separation of chromaticity from lightness can be revealed from the analysis of the dependence of spatial resolution on colour [12,13]. It has been demonstrated that the honeybees [14], budgerigars [15] and some fishes [16,17] have lightness vision that is largely separate from chromatic vision, with lightness having higher spatial resolution than chromaticity. Similar to humans, these animals do not use the short-wavelength photoreceptors for high spatial resolution lightness vision [12–17]. However, some animals, such as goldfish and the hummingbird hawk moths, probably use all photoreceptors for high spatial resolution vision [18,19]. Our spatial vision and detection of borders is predominantly mediated by lightness [20] with chromaticity attributed to spatial location possibly at the late stages of visual processing [21]. Therefore, our reliance on chromaticity for grouping components of patterns cannot be easily explained on the basis of neural processing of colour. The saliency of chromatic cues for grouping of elements can be explained as an adaptation to detection of objects in natural lighting conditions [4]. For primates, lightness does not provide a reliable cue for object segregation in their natural forest habitat, due to the heterogeneous (patchy) light environment [4]. In such conditions, objects can be segregated on the basis of chromatic consistency and, accordingly, humans rely predominantly on chromatic cues for segregating objects [4].

Our ability to recognize shape irrespective of colour and our reliance on chromatic cues for object segregation forms the essence of the Ishihara test for colour deficiency [22]. In this test, people are presented with plates that are composed of scattered dots, which construct target shapes (e.g. numerals) and backgrounds. The dots belonging to a target shape are either consistent in their chromaticity, but have variable lightness, or have variable chromaticity but are consistent in lightness. People with normal colour vision easily detect shapes on the basis of their chromaticity, whereas colour deficient individuals rely predominantly on achromatic cues [22].

In this study, we investigated how fish use colour for the detection and identification of objects and asked if they also segregate different components of potential cues such as chromaticity, lightness and shape. We used the triggerfish (*Rhinecanthus aculeatus*), a reef fish that dwells in shallow marine environments usually close to or on coral reefs, in which illumination is highly contrasting and patchy due to shadows and water surface motion [5]. In such conditions, luminance does not provide a reliable cue for object segregation; therefore, object detection and identification are probably primarily based on chromaticity.

**Figure 1.**
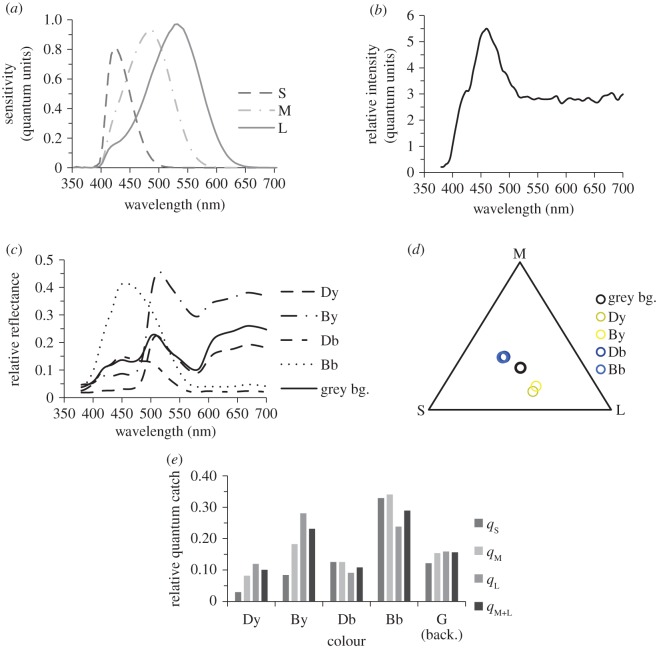
(*a*) Taken from Cheney *et al.* [24], the spectral sensitivities of the S, M and L cones of *R. aculeatus*. (*b*) The within-tank illumination, where the measured reflectance spectra of colours presented on stimuli (*c*) were taken under. (*d*) Maxwell's triangle showing a visualization of colour distance between colour loci (dark yellow, ‘Dy’; bright yellow, ‘By’; dark blue, ‘Db’; and bright blue, ‘Bb’; background, ‘bg’), within the modelled trichromatic colour space of *R. aculeatus*. (*e*) Normalized signal of each colour provided as quantum catches of individual cones (*q*_s_, *q*_M_, *q*_L_) and double cones (summed M and L, *q*_M+L_).

Vision of *R. aculeatus* has been studied in some detail [17,23–26]. This fish is trichromatic and has three spectral types of visual pigments (figure 1*a*), which are housed in single and double cones [23–25]. Single cones contain the short-wavelength (S) pigment while the two members of double cones contain either middle (M)- or long-wavelength (L) pigments [23,24]. It has been demonstrated that *Rhinecanthus aculeatus* can detect a 0.38° dot when it has no contrast for the short-wavelength or middle-wavelength photoreceptors, while the dots that do not have contrast for combined signal of double cones or the long-wavelength cones in order to be detected need to subtend at least 3.3° and 1.7°, respectively [17]. Therefore, *Rhinecanthus aculeatus* uses double cones and possibly the long-wavelength cones for high spatial resolution lightness vision.

## 2. Material and methods

### 2.1. Animals

Wild-caught *R. aculeatus* were collected from shallow reef flats around Lizard Island, Great Barrier Reef, Australia (14°40′8′′ S, 145°27′34′′ E) using hand nets (collecting permits: Great Barrier Reef Marine Park Authority G12/35688 and Queensland Fisheries 161624). Fish ranged in size from 5.7 to 14.5 cm (standard body length, SL) and were individually housed in natural daylight exposed experimental aquaria (see electronic supplementary material, figure S1), at the Lizard Island Research Station. At the end of the study, fish were released at site of original capture. Experiments were conducted under the approval of the University of Queensland's Animal Ethics Committee, approval number: SBS/111/14/ARC.

### 2.2. Stimuli design

Stimuli were constructed using Wolfram Mathematica (10.1), then printed using a Ricoh Aficio MPC4501 onto white (80 gsm) paper, and laminated using a clear sleeve laminator. The reflectance spectra of all colours (figure 1*c*) used in the stimuli were measured using a PR-655 SpectraScan^®^ Spectroradiometer (Photo Research Inc.) under a standard light source (Carl Zeiss, 60 Hz microscope-mounted lamp) relative to a 99% (300–700 nm) white reflectance standard (Ocean Optics). Yellow and blue colours (dark and bright) were chosen because of their known visibility to *R. aculeatus* [24] (figure 1*a*), as well as their common occurrence in reef fish skin patterns [27]. The stimuli were mounted on an achromatic background and experiments were performed under natural illumination in blue tanks (electronic supplementary material, figure S1). The receptor quantum catches (figure 1*e*) were calculated using the illumination measured inside tanks (figure 1*b*). The RGB values of stimuli were adjusted, so that the dark yellow and the bright yellow shared similar chromaticity for fish, as did the dark blue and the bright blue (figure 1*d*). The double cone (M + L), M- and L-cone quantum catches of bright colours differed substantially from those of dark colours (figure 1*e*). On the other hand, the double cone (M + L), M- and L-cone quantum catches of bright blue and yellow were similar to each other, as were the double cone (M + L), M- and L-cone quantum catches dark of yellow and blue colours (figure 1*e*). Because the lightness vision in *R. aculeatus* is mediated by double cones and/or by the L cones [17], bright colours are predicted to be easily discriminated from dark colours on the basis of their lightness. Also, because S cones do not contribute to the *R. aculeatus* lightness vision [17] the bright yellow and bright blue are similar in their lightness, as are the dark yellow and dark blue colours. To quantify the difference between colours, we used the receptor noise limited model [27,28] (for details of calculations see electronic supplementary materials, contrast calculations). Calculations show that all colours used in this study can be discriminated from each other; however, there is a highly salient difference between chromatic properties of yellow and blue and achromatic properties of dark and bright colours (figure 1*d*,*e*; see electronic supplementary material, table S1).

**Figure 2.**
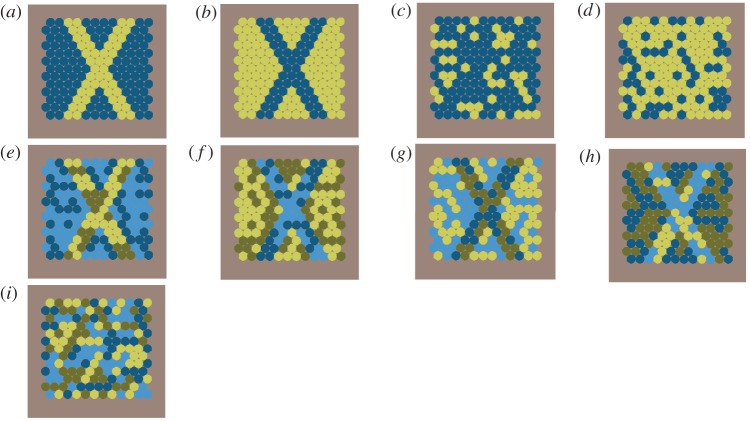
Training stimuli: bright yellow cross and dark blue surround (*a*), dark blue cross and bright yellow surround (*b*), scattered bright yellow distracter (*c*), and scattered dark blue distracter (*d*). Camouflaged stimuli: achromatically camouflaged yellow cross (*e*), achromatically camouflaged blue cross (*f*), chromatically camouflaged blue cross (*g*), chromatically camouflaged yellow cross (*h*), and distracter stimulus with scattered camouflage colours (*i*).

Square-shaped stimuli (5.0 × 5.0 cm) were composed of hexagonally arranged rows of coloured dots presented against a neutral grey background (figure 2). Dots that are 3 mm in diameter have previously been shown to be visible to *R. aculeatus* at a viewing distance of 10 cm [25,29]. Stimuli used during the training phase of the experiment were a cross shape comprising bright yellow dots on a background of dark blue or *vice versa* (figure 2*a*,*b*). In testing, cross stimuli were composed of dots which either had consistent chromaticity, but inconsistent lightness (achromatic noise) (figure 2*e*,*f*) or consistent lightness, but inconsistent chromaticity (chromatic noise) (figure 2*g*,*h*). Visual noise was generated by printing, in addition to the dark blue and bright yellow dots, the bright blue and dark yellow dots in random order. Stimuli with chromatic noise had a random arrangement of blue and yellow dots, with the cross being composed from dots having similar lightness and different chromaticity. Stimuli with achromatic noise had a random arrangement of dark and light dots, with the cross being composed of dots having similar chromaticity and different lightness. Distractor stimuli (figure 2*c*,*d*,*i*) were composed of randomly arranged dots with the same number of dots of a given colour as in the corresponding stimuli with a cross. For each stimulus with randomly arranged dots, we designed 25 replicates using a random number generator (Wolfram Mathematica 10.1). These were presented in a randomly shuffled order each session, to prevent the learning of non-cross related features.

### 2.3. Training

To test the hypothesis that fish can segregate shape on the basis of common colour, we trained *R. aculeatus* via operant conditioning to detect a cross composed of dots presented among a surround of different coloured dots. For the first 5 days in captivity, fish were exposed to the presence of feeding boards in the tank for an hour per day. Fish were initially encouraged to approach cross stimuli by smearing cross stimuli with squid until they approached without food present. Fish were then encouraged to peck stimuli to receive a food reward delivered from above. Group 1 (*n* = 10) were trained to discriminate a bright yellow cross presented against a surround of dark blue dots, from random arrays of dark blue dots and bright yellow dots (figure 2*a*,*c*). Group 2 (*n* = 10) were trained to discriminate a dark blue cross presented against a surround of bright yellow dots, from random arrays of bright yellow and dark blue dots (figure 2*b*,*d*). Stimuli were attached to white acrylic feeding boards (12 × 40 cm), and held 10 cm apart at the presenting tank-end (electronic supplementary material, figure S1). To prevent any side-biases from developing, the position of positive stimuli (left or right) was changed pseudo-randomly between each trial, never being presented on one side for more than three consecutive trials. A choice counted as a single peck anywhere on a single stimulus. A correct choice for the cross-shaped stimulus was immediately rewarded with a small (1–1.5 mm) piece of squid by tweezers from above at the centre of the tank. An incorrect choice went unrewarded and punished by immediate trial termination, with no interaction for 30 s. Stimuli were immediately removed following a choice, to prevent multiple choices being made. Depending on the motivation of a fish, between three and six trials were recorded per session. Most fish learnt the task within 8–12 days and made anywhere between 28 and 62 choices. Fish had successfully learnt the task after reaching a probability threshold of ≥70% correctness held over five consecutive sessions, with five to six trials per session (binomial test, *n* = 28–30, *p *< 0.05).

### 2.4. Experiment

#### 2.4.1. Do fish predominantly use chromatic or achromatic cues when given a direct choice?

After fish had been trained to select the cross stimulus, we performed unrewarded trials where fish had to select between two cross shapes: one camouflaged with achromatic noise (figure 2*e*,*f*), another camouflaged with chromatic noise (figure 2*g*,*h*). These trials were unrewarded to prevent fish from forming a preference for one of the crosses, as this may not be indicative of its saliency. Unrewarded trials were separated by a minimum of three rewarded trials involving training stimuli. Each fish was tested 30 times, over a period of 13–15 sessions.

#### 2.4.2. Do fish predominantly use chromatic or achromatic cues when given an indirect choice?

To further investigate the ability of fish to segregate shape camouflaged by chromatic or achromatic noise, we presented fish with camouflaged crosses (rewarded) (figure 2*e*,*f*), against a random arrangement of dots of different colours (unrewarded) (figure 2*i*). The proportion of dots of each colour in rewarded and unrewarded stimuli was equal. For Group 1 (originally trained using a bright yellow cross), the rewarded stimulus was initially a yellow cross camouflaged with achromatic noise (figure 2*e*) followed by, a bright cross camouflaged with chromatic noise (figure 2*g*). For Group 2 (originally trained using a dark blue cross), the rewarded stimulus was initially a blue cross camouflaged with achromatic noise (figure 2*f*) and then a dark cross camouflaged with chromatic noise (figure 2*h*). All fish conducted a total of 30 choices per set of camouflaged stimuli over a period of five sessions, with the exception of one fish (that conducted *n* = 20 trials) due to an infected fin.

#### 2.4.3. Can fish generalize shape over difference in colour?

To test whether fish generalize shape over difference in colour, we presented fish with a reverse coloured set of stimuli with a distracter stimulus: fish trained with a bright yellow cross (figure 2*a*) (Group 1) were presented with a dark blue cross (figure 2*b*) and those trained with a dark blue cross (Group 2) (figure 2*b*) were presented with a bright yellow cross (figure 2*a*).

### 2.5. Statistical analysis

All statistical tests were conducted using the software package R v. 3.2.2 [30]. All three tests in our experiment were analysed using generalized linear mixed models (GLMM) with a binomial distribution with log link function, from the lmer function in the lme4 package [31]. The outcome (1, correct or achromatically camouflaged stimulus; 0, incorrect or chromatically camouflaged stimulus) was entered as the dependent variable. Rewarded stimuli position (L, left; R, right) and test (chromatic camouflage or achromatic camouflage) were used as fixed factors, and fish identity was a random factor to account for fish being tested multiple times. Analysis was performed separately for fish trained to a bright yellow cross (Group 1) and for fish trained to dark blue cross (Group 2). Initially, the size of the fish (SL) was also included in the model as a covariate, but was found to be insignificant (all models: *p *> 0.73) and subsequently disregarded. Any fish that was found to have a side bias in a test was excluded from that choice analysis, this included one individual in the first test and two individuals in the second test (see electronic supplementary material, tables S2 and S3*a*,*b*).

## 3. Results

### 3.1. Training

**Figure 3.**
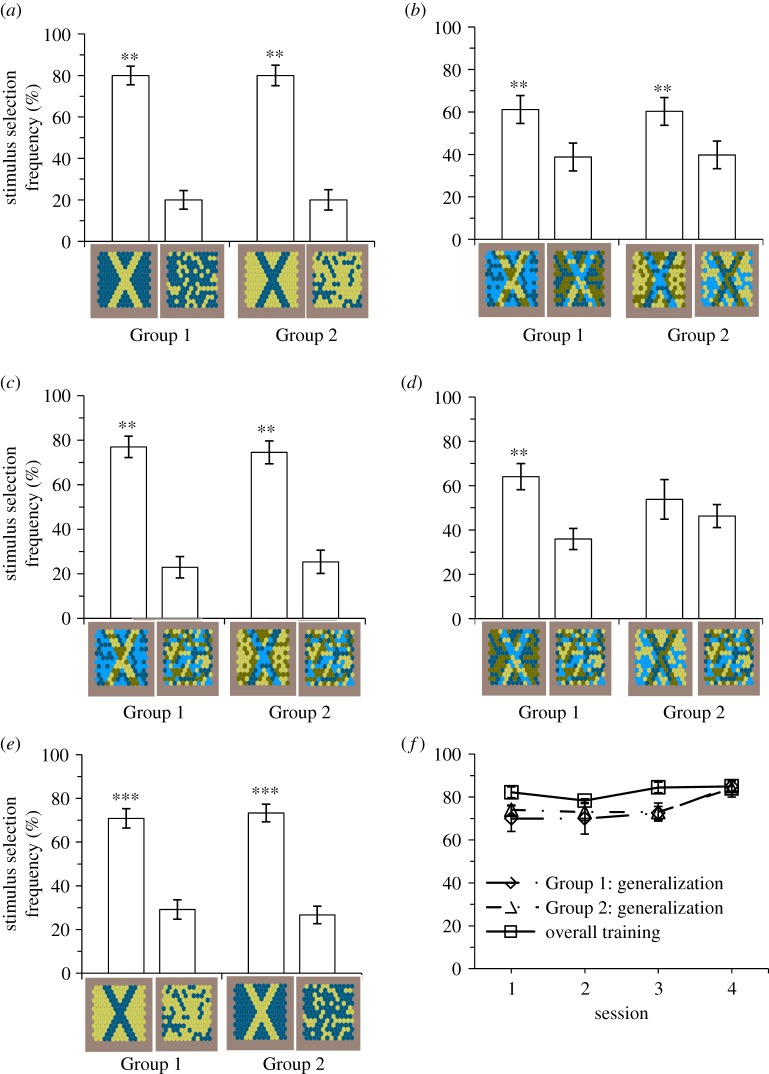
(*a*) Training: Group 1 (*n* = 9 trained to the bright yellow cross) and Group 2 (*n* = 10 trained to the dark blue cross) chose the cross pattern stimulus over the distracter stimulus during training. (*b*) Experiment 1: After training, fish (Group 1: *n* = 8, Group 2: *n* = 10) chose achromatically camouflaged crosses more frequently than crosses that were chromatically camouflaged. (*c*) and (*d*) Experiment 2: The chromatic cross was reliably detected when presented alongside a distracter stimulus (*c*) by both Group 1 (*n* = 9) and Group 2 (*n* = 8). Group 1 also reliably detected the achromatic cross from a distracter stimulus (*d*). (*e*) and (*f*) Experiment 3: Group 1 (*n* = 8), and Group 2 (*n* = 10) reliably detected a novel coloured cross from a distracter stimulus (*e*) during the generalization task. A comparison between the performance of the generalization task and the final four pre-experimental sessions (that involved training stimuli) (*f*), shows little difference in the total number of correct choices across each session. Bars represent mean values, and error bars are ± 1 s.e., ‘**’ denotes statistical significance (*p *< 0.05), ‘***’ denotes statistical significance (*p *< 0.001).

All, but one fish from Group 1 learnt to detect the cross shape with a minimum of 70% correct choices (figure 3*a*; for individual performance of fish during training see electronic supplementary material, figure S2). This indicated that fish could group dots into a shape on the basis of common chromaticity and/or lightness. The fish that did not learn the task was dropped from the experiment. There was no difference between Group 1 (yellow cross; *n* = 9) and Group 2 (blue cross; *n* = 10) in the overall performance during the last 5 session of training, i.e. in their ability to learn the task (GLMM; binomial: *z* = −0.47, *n* trials = 753, *n* fish = 19, *p* = 0.635; figure 3*a*).

#### 3.1.1. Do fish predominantly use chromatic or achromatic cues when given a direct choice?

When presented with a chromatically camouflaged cross (similar lightness and different colour) and an achromatically camouflaged cross (similar colour and different lightness), fish from both training groups were significantly more likely to choose the chromatic cross (figure 3*b*, Group 1, GLMM; binomial: *z* = 2.87, *n* fish = 8, *n* trials = 240, *p *< 0.01; Group 2: *z* = 3.45, *n* fish = 10, *n* trials = 300, *p *< 0.001; for individual performance see electronic supplementary material, table S2). This suggests that fish relied more heavily on chromatic cues for object segregation.

#### 3.1.2. Do fish prefer chromatic or achromatic cues when given an indirect choice?

When fish were presented with a chromatic cross and a distracter stimulus, they were significantly more likely to choose crosses than the distracter stimulus (figure 3*c*, chromatic cross; Group 1, GLMM; binomial: *z* = 5.54, *n* fish = 9, *n* trials = 260, *p *< 0.001; Group 2, *z* = 4.88, *n* fish = 8, *n* trials = 240, *p *< 0.001). Only fish in Group 1 were also found to be significantly more likely to choose achromatic crosses over the distracter stimulus (figure 3*d*, achromatic cross; Group 1, GLMM; binomial: *z* = 3.67, *p *< 0.001; Group 2, *z *= 0.56, *p *= 0.576). Additionally, fish made significantly more correct choices when choosing between chromatic crosses compared with achromatic crosses (Group 1, GLMM; binomial: *z* = 7.01, *n* trials = 520, *p *< 0.01; Group 2: *z* = 5.58, *n* trials = 480, *p *< 0.001). These results suggest that fish were able to detect crosses camouflaged by both achromatic and chromatic noise, and that it was easier for fish to segregate crosses based on chromaticity than on lightness.

#### 3.1.3. Can fish generalize shape over difference in colour?

When fish were presented with stimuli of reversed colour from those presented in training, they were significantly more likely to make a correct choice for the cross-shape stimulus compared with the distracter stimulus (figure 3*e*, Group 1, GLMM; binomial: *z* = 4.80, *n* fish = 8, *n* trials = 240, *p *< 0.001; Group 2: *z* = 5.83, *n* fish = 10, *n* trials = 300, *p *< 0.001). The level of performance during this generalization test was also similar to the initial training for at least 14 out of the 18 fish (five failed to discriminate, see electronic supplementary material, table S4). For both Groups 1 and 2, performance during the first session of the generalization test (figure 3*f*) was very similar to the estimated overall level of performance [mean (%) ± s.e. = 75.3 ± 2.8], as well as to the overall level of performance for the final four pre-experimental sessions with training stimuli [mean (%) ± s.e. = 82.5 ± 2.0]. It seems most fish generalized the shape of the training stimulus over its colour, rather than relearned the novel stimulus.

## 4. Discussion

We have shown that *R. aculeatus* groups dots to segregate shape and generalizes shape irrespective of colour and lightness. These findings support the hypothesis of a similarity of object detection strategies among different animals and humans.

*R. aculeatus* was able to detect the cross shape when it was camouflaged both with chromatic and achromatic noise. However, fish were better at distinguishing the cross when it was chromatically consistent compared with crosses with chromatic variability/noise. From this, we conclude that *R. aculeatus* relied more heavily on chromatic cues for object segregation. Previous studies have demonstrated that a number of animals including the honeybee (*Apis mellifera*) [14], birds [15,32] and humans [33] mainly rely on lightness (achromatic) cues for detecting and discriminating shape and small targets, while chromatic cues are primarily used for discriminating colours of stimuli subtending large visual angles. A recent study also demonstrated that *R. aculeatus* chose stimuli based on achromatic cues rather than chromatic cues when viewing small stimuli [29]. Newport *et al.* also found that *R. aculeatus* learnt larger stimuli via chromaticity, rather than pattern/shape or luminance/lightness [29]; however, in their study, rewarded conspicuous stimuli were discriminated against similarly conspicuous distracter stimuli. The present study is a detection task, rather than a discrimination task, therefore different results may be expected as discrimination tasks require memory of specific objects, whereas detection tasks do not [13].

The importance of chromatic cues for object segregation can be explained as an adaptation to detection and identification of objects in conditions of spatially and temporally variable illumination. Spatial variation of illumination renders lightness unreliable and, hence, chromaticity becomes a more stable cue for segregation of object shape [4,6]. The patchiness of illumination is characteristic of forest habitat and it has been proposed that variations of lighting conditions explains the greater weight given to chromatic signals by primates [4] and other forest-dwelling species [34–36]. In shallow aquatic environments, wave motion produces patchy illumination, which may explain the usefulness of chromatic cues for segregation of objects for reef fish. However, whether fishes that dwell in habitats at greater depths with uniform illumination [37] rely on chromaticity for object segregation, remains unclear.

Our conclusion that chromaticity is a dominant cue for object segregation is based on comparing extreme chromatic contrast (yellow–blue) to extreme achromatic contrast (bright–dark). Both differences correspond to the range of 30–50 just noticeable differences (JNDs) (electronic supplementary material, table S1) and are probably close to the saturation of the saliency of contrasts. Generally, animals rely on a more salient cue and, therefore, in the case of unsaturated colours strong achromatic contrast is likely to be more salient than chromatic contrast. It would be interesting to investigate how the ability of fish to segregate objects depends on the relative amounts of chromatic and achromatic contrast, and how the saliency of contrast depends on chromatic and achromatic contrasts. For humans, chromaticity and lightness belong to different modalities; therefore, comparing the two on the same scale is a difficult task [38]. Human observers can make reliable pair-wise contrast matches between gratings that differ along chromatic and achromatic axes [38]. Aside from previous behavioural work on the vision of the honeybee [39] and crow [40], the comparison of the saliency of chromatic and achromatic cues of different contrasts for shape segregation has not yet been performed for humans, nor for most animals.

Similar to primates, fish generalize shape over colour, which probably helps in the recognition of objects when colour changes depend on illumination, viewing angle and distance to object [41]. The fact that fish generalize shape over colour, suggests that similar to humans, fish also process colour and shape separately. In humans, the separation of colour and shape is achieved by independent processing of different aspects of visual stimuli in the visual cortex [7].

Previous studies have shown that fish and other animals are capable of performing tasks that are thought to require complex cortical processing in humans. For example, archerfish can recognize faces [42] and various species of fish amodaly complete objects [43,44]. Fish can also be tricked by optical illusions including the Ebbinghaus illusion [45], illusory motion [46] and lightness illusion [46]. Additionally, fishes and bees have been found to perceive illusory contours [47–49]. The ability of animals to carry out complex visual tasks and perceive visual illusions, which we also perceive, supports the hypothesis that fish and other ‘lower animals’, including insects, use similar neural strategies for object detection and discrimination. However, since ‘lower animals’ do not have a visual cortex, the neural implementation of these ‘algorithms’ in fish may be more down-stream, even starting with the retina [50,51].

Natural lighting conditions have a strong influence over which visual cues are most salient to observers. *R. aculeatus* appears to depend more on chromatic cues for object segregation and this may be due to the presence of high achromatic noise in shallow marine habitats. However, our conclusion is derived using extreme chromatic difference between colours. Further investigation involving a range of intermediate colours with less extreme chromatic contrast and greater differences in intensity is necessary to fully understand the importance of chromatic and achromatic cues. Finally, the non-cortical structures in fish that are responsible for independently processing the visual signals of colour and shape seem to exhibit a similar neural strategy implemented by relevant cortical structures found in humans.

## Supplementary Material

Contrast Calculations

## Supplementary Material

Supplementary Fig 1

## Supplementary Material

Supplementary Fig 2

## Supplementary Material

Supplementary Tables

## Data accessibility

This study's dataset is available at Dryad: https://doi.org/10.5061/dryad.dm133 [52].
