# Supplementary material for: Triggerfish uses chromaticity and lightness for object segregation
Source: R Soc Open Sci. 2017 Dec 20;4(12):171440. doi: 10.1098/rsos.171440 (PMC5750034; doi:10.1098/rsos.171440)
Supplement: Contrast Calculations [file rsos171440supp1.docx]

**Quantum catches equation (following Vorobyev & Osorio, 1998):**

Cone signals (*q_i_*) were calculated as relative quantum catches by

*q_i_* = *k_i_* ∫ *R_i_*(*λ*) *I*(*λ*) *S*(*λ*) d *λ* ,

where wavelength is denoted as *λ*, *R_i_*(*λ*) is the spectral sensitivity of a receptor type *i* (*i* = L, M, S), *I*(*λ*) is the within-tank illumination spectrum (Fig. S2c), and *S*(*λ*) is the reflectance spectra of the coloured stimuli, with integration across the visible spectrum (Vorobyev & Osorio, 1998). The scaling factor, *k*_i_ has a chosen value so that the quantum catch of an ideal white reflectance standard is equal to 1, where

*k*_i_ = 1 ∕ ∫ *R_i_*(*λ*) *I*(*λ*) *S*(*λ*) d *λ*

**Formulae for calculating colour distance (Δ*S*^2^) (taken from Vorobyev *et al.* 2001):**

By following the log-linear receptor noise-limited model (Vorobyev *et al.* 2001), it was possible to calculate receptor signals (*f_i_*) using the formula

*f_i_* = ln(*q_i_*)

A chromaticity diagram was then constructed to visually plot spectra within a colour space, where the Euclidean distance between points indicated the predicted ability of colour discrimination. This diagram followed the same design as that shown in Hempel de Ibarra, Giurfa & Vorobyev (2001), with axes that corresponded identically to the L - M (X_1_) and S - [L + M] (X_2_) gradients:

$X_{1}=A\left( f_{L}- f_{M} \right),$

$X_{2}= B\left( f_{S} -\left( {af}_{L} +{bf}_{M} \right) \right),$

Where:

*A* = $\sqrt{\frac{1}{({\omega_{M})}^{2}+ ({\omega_{L})}^{2}}}$,

*B* = $\sqrt{\frac{({\omega_{M})}^{2}+ ({\omega_{L})}^{2}}{{(\omega_{S}\omega_{M})}^{2}+ {(\omega_{S}\omega_{L})}^{2}+ {(\omega_{M}\omega_{L})}^{2}}}$,

$a= \frac{{{(\omega}_{M})}^{2}}{{{(\omega}_{M})}^{2}+ {{(\omega}_{L})}^{2}}$,

$b= \frac{{{(\omega}_{L})}^{2}}{{{(\omega}_{M})}^{2}+ {{(\omega}_{L})}^{2}},$

Noise values (*ω_i_*) were input as, *ω*_S_ = 0.05, *ω*_M_ = 0.022 and *ω*_L_ = 0.022 (Champ, 2014). Estimates of colour distance (Δ*S*^2^) were calculated by

${\Delta S}^{2}= {\Delta X}_{1}^{2}+ {\Delta X}_{2}^{2}$

Achromatic cone specific contrasts were calculated as

$$\Delta S_{achrom}=\frac{\Delta f_{i}}{\omega_{i}},$$

where *i* corresponds to the spectral type of cone.
