# Supplementary material for: Triggerfish uses chromaticity and lightness for object segregation
Source: R Soc Open Sci. 2017 Dec 20;4(12):171440. doi: 10.1098/rsos.171440 (PMC5750034; doi:10.1098/rsos.171440)
Supplement: Supplementary Fig 1 [file rsos171440supp2.pdf]

(a)

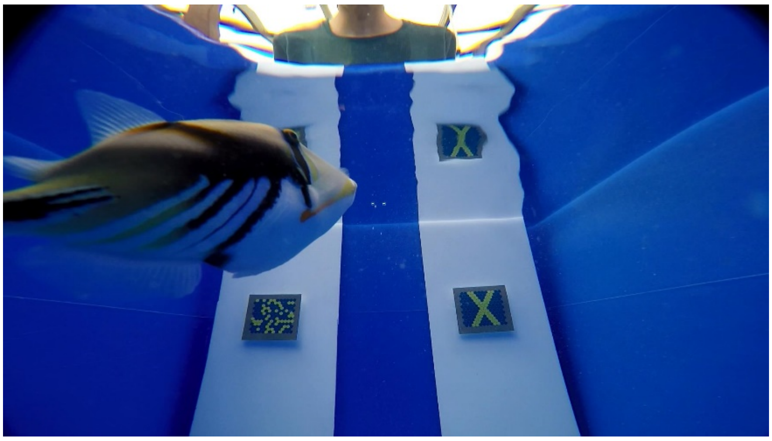

(b)

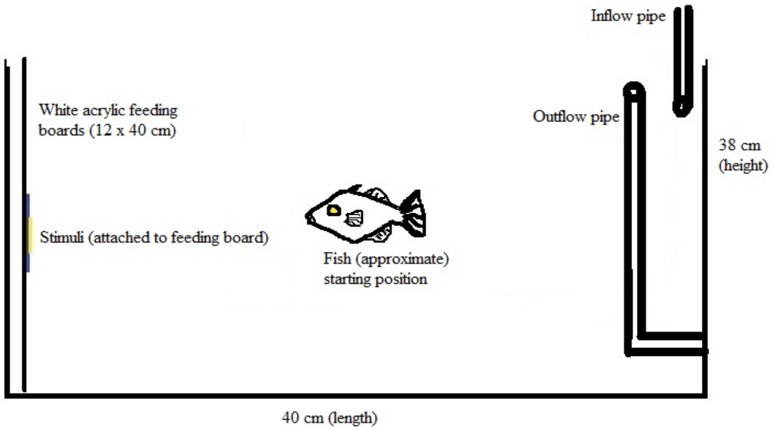

Supplementary Figure. 1. Within-tank view of presenting tank-end (a). Note: the PVC hide was removed, and air pump was switched off during both training and experiments. Illustration of experimental aquaria (b) showing the approximate starting position of fish relative to presented stimuli.
