# Supplementary material for: Triggerfish uses chromaticity and lightness for object segregation
Source: R Soc Open Sci. 2017 Dec 20;4(12):171440. doi: 10.1098/rsos.171440 (PMC5750034; doi:10.1098/rsos.171440)
Supplement: Supplementary Fig 2 [file rsos171440supp3.pdf]

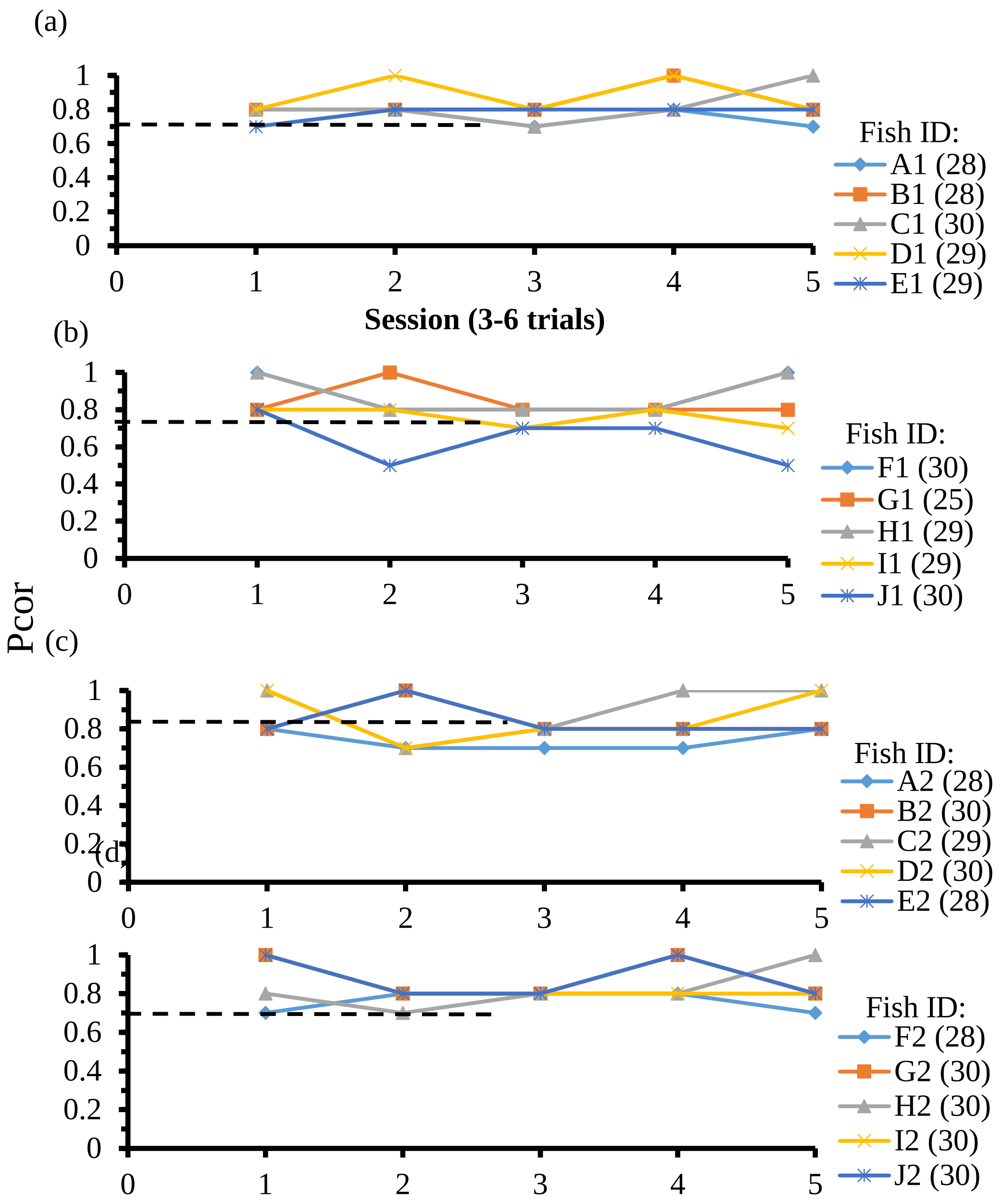

Supplementary Figure. 2. Training session performance with proportion of correct choices (Pcor) recorded for individuals (Fish ID) from fish which learnt the yellow cross (a and b) and fish which learnt the blue cross (c and d). The dashed-line marks a learning threshold of 0.7 correct choices, and the total number of choices made by each fish is enclosed in parentheses. Fish J1 was the only individual that failed to learn the cross stimulus.
