# Supplementary material for: Triggerfish uses chromaticity and lightness for object segregation
Source: R Soc Open Sci. 2017 Dec 20;4(12):171440. doi: 10.1098/rsos.171440 (PMC5750034; doi:10.1098/rsos.171440)
Supplement: Supplementary Tables [file rsos171440supp4.pdf]

Colour	q_S	q_M	q_L	q_{M+L}	X1, X2
DY	0.03	0.08	0.12	0.10	12.18, -33.98
BY	0.08	0.18	0.28	0.23	13.81, -28.05
DB	0.13	0.13	0.09	0.11	-10.37, 4.48
BB	0.33	0.34	0.24	0.29	-11.51, 4.12
G (back.)	0.12	0.15	0.16	0.16	1.13, -7.15
**ΔS_{achrom}(intra.pair)**	**S**	**M**	**L**	**M+L**	**ΔS_{chrom}(intra.pair)**
DY	20.75	36.47	38.79	37.86	6.15
BY	-	-	-	-	-
DB	19.33	45.32	43.71	44.65	1.20
BB	-	-	-	-	-
**ΔS_{achrom}(inter.pair)**					**ΔS_{chrom} (inter.pair)**
DB-DY	28.76	19.48	-12.4	3.321	44.59
DB-BY	8.01	-17	-51.2	-34.5	40.54
BB-DY	48.09	64.81	31.31	47.97	44.87
BB-BY	27.34	28.33	-7.48	10.11	40.95
**ΔS_{achrom}(back.)**					**ΔS_{chrom}(back.)**
DY	-28.15	-28.67	-13.05	-20.06	29.02
BY	-7.40	7.80	25.74	7.83	24.45
DB	0.61	-9.19	-25.45	-16.74	16.35
BB	19.94	36.13	18.26	27.91	16.94

Supplementary Table 1. Quantum catches of individual cones (q_s, q_M, q_L) and double cones (q_{M+L}) , chromatic coordinates (X_1, X_2), chromatic (ΔS_{chrom}) and receptor specific achromatic (ΔS_{achrom}) colour differences for all colours presented on stimuli [dark yellow (DY), bright yellow (BY), dark blue (DB), bright blue (BB), and the grey background (G)]. For details of calculations see supplementary materials.

Fish ID	Left	Right	Significance (*P* < 0.05)	chromatic	achromatic	*P* chrom	95% CI	Significance (*P* < 0.05)
A1	14	16	0.855	14	16	0.47	0.283 - 0.657	0.856
B1	13	17	0.585	17	13	0.57	0.374 - 0.745	0.585
C1	11	19	0.201	22	8	0.73	0.541 - 0.877	**0.0161**
D1	14	16	0.856	20	10	0.67	0.454 - 0.808	0.15
E1	9	21	**0.043**	-	-	-		-
F1	14	16	0.856	19	13	0.59	0.406 - 0.763	0.201
G1	17	13	0.585	24	6	0.80	0.614 - 0.923	**0.0143**
H1	14	16	0.856	17	13	0.57	0.374 - 0.745	0.585
I1	15	15	1	15	15	0.50	0.313 - 0.687	1
A2	15	15	1	21	9	0.70	0.506 - 0.853	**0.0428**
B2	17	13	0.585	17	13	0.57	0.374 - 0.745	0.585
C2	9	4	0.267	9	4	0.69	0.386 - 0.909	0.267
D2	14	16	0.856	17	13	0.57	0.374 - 0.745	0.585
E2	18	12	0.362	18	12	0.60	0.264 - 0.623	0.597
F2	12	18	0.362	20	10	0.67	0.500 - 0.839	**0.0501**
G2	11	19	0.201	17	13	0.57	0.255 - 0.626	0.585
H2	13	17	0.585	15	15	0.50	0.313 - 0.687	1
I2	16	14	0.856	21	9	0.70	0.506 - 0.853	**0.0428**
J2	14	16	0.467	14	16	0.47	0.283 - 0.657	0.856

Supplementary Table. 2. Shown is the number of choices made by individual fish (Fish ID) for “chromatic” cross pattern stimuli, and “achromatic” cross pattern stimuli, as well as the proportion of choices made for chromatic cross stimuli (*P*chrom), 95% binomial confidence intervals (95% CI) and the statistical significance (P-value). Rewarded stimuli were presented equally often on the right and left side. Side bias analysis (left or right) found that only fish E1 made significantly more choices for right-hand positioned stimuli. Values of significance in bold indicate a less than 0.05 probability (binomial test) that a fish chose equally between chromatic and achromatic cross stimuli, exceeding a 0.5 chance of no effect.

(a)

Fish ID	Left	Right	Significance (p < 0.05)	Correct	Incorrect	Pcorr	95% CI	Significance (p < 0.05)
A1	17	13	0.585	23	7	0.77	0.577 - 0.901	**0.00522**
B1	17	13	0.585	20	10	0.67	0.472 - 0.827	0.0987
C1	15	15	1	18	12	0.60	0.406 - 0.773	0.3616
D1	16	14	0.856	17	13	0.57	0.374 - 0.745	0.5847
E1	15	15	1	20	10	0.67	0.472 - 0.827	0.0987
F1	14	16	0.856	17	13	0.57	0.374 - 0.745	0.585
G1	15	15	1	19	11	0.63	0.439 - 0.801	0.201
H1	16	14	0.856	17	13	0.57	0.374 - 0.745	0.585
I1	14	16	0.856	19	11	0.63	0.439-0.801	0.201
A2	16	14	0.856	23	7	0.77	0.577 - 0.901	**0.00522**
B2	12	18	0.3616	17	13	0.57	0.374 - 0.745	0.585
C2	16	14	0.856	19	11	0.63	0.439 - 0.801	0.201
D2	17	13	0.585	10	20	0.33	0.173 - 0.528	0.0987
E2	16	14	0.856	13	17	0.43	0.255 - 0.626	0.585
F2	16	14	0.856	13	17	0.43	0.255 - 0.626	0.585
G2	15	15	1	17	13	0.57	0.374 - 0.745	0.585
H2	7	23	**0.00522**	-	-	-	-	-
I2	10	20	0.0987	17	13	0.57	0.374 - 0.745	0.585
J2	5	25	**0.000325**	-	-	-	-	-

(b)

Fish ID	Left	Right	Significance (p < 0.05)	Correct	Incorrect	Pcorr	95% CI	Significance (p < 0.05)
A1	14	16	0.856	25	5	0.83333	0.283 - 0.657	**0.000325**
B1	14	16	0.856	23	7	0.76667	0.577 - 0.901	**0.00522**
C1	13	17	0.585	24	6	0.8	0.614 - 0.923	**0.00143**
D1	16	14	0.856	25	5	0.83333	0.653-0.944	**0.000325**
E1	15	15	1	17	13	0.56667	0.374 - 0.745	0.585
F1	15	15	1	24	6	0.8	0.614 - 0.923	**0.00143**
G1	18	12	0.362	20	10	0.66667	0.472 - 0.827	0.0987
H1	11	9	0.824	18	2	0.9	0.683 - 0.988	**0.000403**
I1	16	14	0.856	23	7	0.76667	0.577 - 0.901	**0.00522**
A2	17	13	0.585	24	6	0.8	0.614 - 0.923	**0.00143**
B2	16	14	0.856	22	8	0.73333	0.541 - 0.877	**0.0161**
C2	16	14	0.856	21	9	0.7	0.506 - 0.853	**0.0428**
D2	17	13	0.585	22	8	0.73333	0.541 - 0.877	**0.0161**
E2	15	15	1	23	7	0.76667	0.577 - 0.901	**0.00522**
F2	16	14	0.856	22	8	0.73333	0.541 - 0.877	**0.0161**
G2	17	13	0.585	23	7	0.76667	0.577 - 0.901	**0.00522**
H2	-	-	-	-	-	-	-	-
I2	17	13	0.585	22	8	0.73333	0.541 - 0.877	**0.0161**
J2	-	-	-	-	-	-	-	-

Supplementary Table. 3. Shown is the number of ("correct" and "incorrect") choices and proportion of correct choices (Pcorr) that individual fish (Fish ID) made when discriminating cross pattern stimuli (correct choice) from non-cross stimuli (incorrect choice). Cross pattern stimuli were camouflaged by either **a)** chromatic noise (achromatic cross), or **b)** achromatic noise (chromatic cross). Side bias analysis for left- and right-hand positioned stimuli found that fish H2 and fish J2 had significant bias for right-hand positioned stimuli when tested with achromatic cross stimuli **(a)**. Both of the individual's results were disregarded in the data analysis. Values of significance in bold indicate a less than 0.05 probability that a fish chose equally between achromatic/chromatic cross stimuli and distracter stimuli, exceeding a 0.5 chance of no effect. *P*-values and 95% binomial confidence intervals (95% CI) were acquired from binomial tests.

Fish ID	Left	Right	Significance ($p < 0.05$)	Correct	Incorrect	Pcorr	95% CI	Significance ($p < 0.05$)
A1	15	15	1	23	7	0.77	0.577 - 0.901	**0.00522**
B1	17	13	0.585	24	6	0.80	0.614 - 0.923	**0.00143**
C1	14	16	0.856	24	6	0.80	0.614 - 0.923	**0.00143**
D1	19	11	0.201	15	15	0.50	0.312 - 0.687	1
E1	14	16	0.856	20	10	0.67	0.472 - 0.827	0.0987
F1	16	14	0.856	24	6	0.80	0.614 - 0.923	**0.00143**
G1	17	13	0.585	19	11	0.63	0.439 - 0.801	0.201
H1	-	-	-	-	-	-	-	-
I1	13	17	0.585	21	9	0.70	0.506 - 0.853	**0.0428**
A2	16	14	0.856	23	7	0.77	0.577 - 0.901	**0.00522**
B2	16	14	0.856	24	6	0.80	0.614 - 0.923	**0.00143**
C2	13	17	0.585	23	7	0.77	0.577 - 0.901	**0.00522**
D2	16	14	0.856	23	7	0.77	0.577 - 0.901	**0.00522**
E2	16	14	0.856	20	10	0.67	0.472 - 0.827	0.0987
F2	15	15	1	20	10	0.67	0.472 - 0.827	**0.00522**
G2	18	12	0.362	22	8	0.73	0.541 - 0.877	**0.0161**
H2	14	16	0.856	21	9	0.70	0.614 - 0.923	**0.0428**
I2	19	11	0.201	23	7	0.77	0.577 - 0.901	**0.00522**
J2	14	16	0.856	21	9	0.70	0.614 - 0.923	**0.0428**

Supplementary Table. 4. Shown is the number of ("correct" and "incorrect") choices, and proportion of correct choices (Pcorr) that individual fish (Fish ID) made when discriminating cross pattern stimuli (from distracter stimuli) which had a reverse colour scheme to that viewed during training. Fish H1 refused to make any choices during the test. No individual was detected to have a significant side bias for left- or right-hand positioned stimuli. Values of significance in bold indicate a less than 0.05 probability that a fish chose equally between cross stimuli and distracter stimuli, exceeding a 0.5 chance of no effect. *P*-values and 95% binomial confidence intervals (95% CI) were acquired from binomial tests.
